# Computational immune synapse analysis reveals T-cell interactions in distinct tumor microenvironments

**DOI:** 10.21203/rs.3.rs-2968528/v1

**Published:** 2023-06-01

**Authors:** Victor Wang, Zichao Liu, Jan Martinek, Jie Zhou, Hannah Boruchov, Kelly Ray, Karolina Palucka, Jeffrey Chuang

**Affiliations:** National Institutes of Health; 1The Jackson Laboratory for Genomic Medicine; The Jackson Laboratory for Genomic Medicine; The Jackson Laboratory for Genomic Medicine; The Jackson Laboratory for Genomic Medicine; The Jackson Laboratory for Genomic Medicine; The Jackson Laboratory for Genomic Medicine; The Jackson Laboratory

**Keywords:** tumor microenvironment, immunological synapse, melanoma, breast cancer, multiplex imaging, imaging mass cytometry

## Abstract

The tumor microenvironment (TME) and the cellular interactions within it can be critical to tumor progression and treatment response. Although technologies to generate multiplex images of the TME are advancing, the many ways in which TME imaging data can be mined to elucidate cellular interactions are only beginning to be realized. Here, we present a novel approach for multipronged computational immune synapse analysis (CISA) that reveals T-cell synaptic interactions from multiplex images. CISA enables automated discovery and quantification of immune synapse interactions based on the localization of proteins on cell membranes. We first demonstrate the ability of CISA to detect T-cell:APC (antigen presenting cell) synaptic interactions in two independent human melanoma imaging mass cytometry (IMC) tissue microarray datasets. We then generate melanoma histocytometry whole slide images and verify that CISA can detect similar interactions across data modalities. Interestingly, CISA histoctyometry analysis also reveals that T-cell:macrophage synapse formation is associated with T-cell proliferation. We next show the generality of CISA by extending it to breast cancer IMC images, finding that CISA quantifications of T-cell:B-cell synapses are predictive of improved patient survival. Our work demonstrates the biological and clinical significance of spatially resolving cell-cell synaptic interactions in the TME and provides a robust method to do so across imaging modalities and cancer types.

## Introduction

The immune system is a major component of the tumor microenvironment (TME) and plays a central role in the modern approach to oncology. The TME has been the target of recent therapeutics that increase the activity of the immune system ^1^, improving survival for patients with tumors including metastatic melanoma ^2^. Pre-existing anti-tumor immunity has long been a prognostic factor in patient survival ^3,4^ and has been associated with response to immune checkpoint blockade ^5,6^. Conversely, absent or dysfunctional anti-tumor immunity generally leads to poor survival and response to treatment ^7,8^. Better parsing of the TME factors and mechanisms mediating anti-tumor immunity is therefore critical to the improvement of immunotherapy.

Cellular interactions within the TME modulate the immune response, often suppressing anti-tumor immunity and worsening patient survival ^9^. T-cells are of particular interest owing to their ability to eliminate cancer cells ^10^ and as key mediators in the immune checkpoint blockade pathway ^11–14^. Immunosuppressive interactions targeting T-cells can prevent their infiltration into the TME, dampen anti-tumor functions, and lead to worse outcomes ^7^, and tumor-resident cells such as dendritic cells may also play a role in T-cell-mediated anti-tumor immunity ^15^. Further study to understand T-cell-TME interactions and their clinical implications is an important need.

A central aspect of T-cell interaction and function Is the immune synapse (IS). The IS forms following recognition of antigens on antigen-presenting cells (APCs), triggering a molecular cascade where the T-cell receptor (TCR) and other signaling molecules aggregate at the point of contact with the APC ^16^. This process is initially required for T-cell activation by professional APCs where T-cells are primed against non-self antigen ^17,18^. Once primed, IS formation is necessary for and precedes acquired immunity functions such as delivering a cytotoxic payload to target (e.g. cancer) cells or triggering the release of cytokines ^19,20^. Thus, IS formation is indicative of T-cell antigen recognition and induction of its functional capabilities. Recent studies have described important new T-cell behaviors by curating examples from image data, e.g. by using lattice light sheet microscopy to show macrophages with exhausted CD8 T-cells ^21^ or by using 3D CyCIF to show CD8 T-cells with melanoma cells ^22^. However, such approaches are limited by manual selection, and automated quantification would be beneficial.

Advances in tumor imaging provide new opportunities to understand immune synapses, but new analysis methods are needed. Proteomic imaging platforms can simultaneously measure dozens of epitopes at subcellular resolution within tumor samples ^23^, and a number of groups have harnessed multiplex imaging methods to associate spatial distribution patterns of immune cells with patient survival or therapy response ^24–36^, typically realized through analysis of cell-cell colocalization or cellular feature intensities. However, approaches based on subcellular information, such as the formation of immune synapses, remain little explored. New methods to automatically evaluate IS formation thus could be valuable to improve T-cell mechanistic quantification and discovery of TME-related biomarkers.

In this work we introduce a computational framework to investigate immune synapses in the TME, which we refer to as *Computational Immune Synapse Analysis* (CISA). We first assess its performance in capturing relevant T-cell IS biology and then demonstrate its effectiveness in identifying functionally relevant cell interactions from high-resolution multiplex TME imaging data across multiple imaging modalities and datasets. Our findings demonstrate the capability and value of interrogating computationally-defined immune synapse formation from imaging data to evaluate biologically and translationally relevant interactions in the *in situ* TME.

## Results

### Image-Based Computational Immune Synapse Analysis

We hypothesized that immune synapses between cells can be detected based on the preferential localization of proteins at interfaces where cells contact one another. While this concept is intuitive, it has been unknown whether current spatial proteomic profiling technologies, such as imaging mass cytometry, have sufficient resolution and precision to reveal such synapses. It also remains poorly understood what fraction of cell-cell contacts will have active synapses in a given tissue sample, as this quantity will depend on the cell types of interest, cell densities, the strength and persistence of synapses, and the tissue type. In addition, synapse quantification is affected by the accuracy of cell segmentation algorithms in identifying cell-cell interfaces in 2D spatial proteomic images. Given these uncertainties, a statistical approach that not only evaluates individual cell-cell contacts, but also integrates across an image, would be valuable to quantifying synaptic activity.

**Figures 1:**
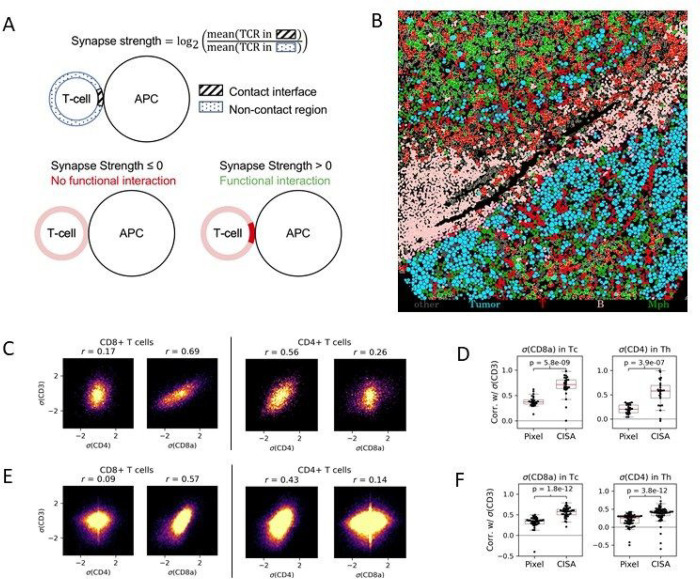
Computational Immune Synapse Analysis. (A) Synapse strength s is the logarithm of the mean membrane signal intensity of CD3 at the T-cell-APC contact interface relative to the intensity outside the contact interface. Synapse strengths < 0 indicate a lack of interaction; synapse strengths > 0 indicate an active interaction. (B-D) are based on melanoma IMC spots from Moldoveanu et al.35 (B) Example of segmented cells. (C) Left to right: Density plots showing correlation of CD4 CISA score with CD3 CISA score in CD8+ cells; correlation of CD8 CISA with CD3 CISA in CD8+ cells; correlation of CD4 CISA with CD3 CISA in CD4+ cells; correlation of CD8 CISA with CD3 CISA in CD4+ cells. (D) Left: Comparison of CD8/CD3 correlation in CD8+ cells at the pixel level to CISA. Right: Comparison of CD4/CD3 correlation in CD4+ cells to CISA. (E,F) Plots are analogous to (C,D) but for melanoma IMC data from Hoch et al.34

To interrogate cell-cell interactions in the *in situ* immunological context, we defined an image-based T-cell immune synapse metric. T-cells are known to interact with antigen presenting cells (APCs), which motivated us to focus on the behavior of TCR proteins at interfaces between T-cells and APCs; however, the approach we describe here is generalizable to any cell types and synapse markers. A T-cell’s synapse strength σ is defined as the logarithm of the mean CD3 signal intensity (a proxy for TCR signal) at the membrane region in contact with an antigen presenting cell (APC) divided by the mean signal in the noncontact region (Fig. 1A). A positive synapse strength indicates T-cell synapse formation and potential functional interaction with another cell in the TME, modeling how the TCR aggregates at the synapse with an APC upon recognition of the presented antigen ^16^. The synapse strength σ can also be considered for each tissue sample, by averaging the values of σ for all contacts between T-cells and APCs (or specified cell type) in the sample. We also implemented a null synapse model based on random sets of contiguous T-cell membrane pixels to account for baseline CD3 aggregation (see Methods). This *computational immune synapse analysis* (CISA) provides a way to investigate the functional importance of cell-cell contacts from images.

To test whether CISA is capable of quantifying immune synapses in high-resolution multiplexed images, we first applied it to a published melanoma IMC dataset from Moldoveanu et al.^35^ This dataset contains 30 pre-treatment patient tissue microarray samples from melanoma patients that received immune checkpoint inhibition therapy. We segmented cells in each image and mapped to the cell annotations from Moldoveanu et al^35^. (see Methods), shown for example in Fig. 1B. We then used CISA to compute $\sigma_{CD3}\left(\;\text{T-CD8}+,\;APC\right)$ for each T-cell:APC contact, i.e. the relative localization of CD3 to the contact region between a CD8 + T-cell and an adjacent APC. We also computed $\sigma_{CD8}\left(\;\text{T-CD8}+,\;APC\right)$, the localization of CD8 toward the adjacent APC. We observed strong correlation (Fig. 1C) in the directional enrichment of CD3 and CD8 toward adjacent APCs, with r=0.69 between $\sigma_{CD3}\left(\;\text{T-CD8}+,\;APC\right)$ and $\sigma_{CD8}\left(\;\text{T-CD8}+,\;APC\right)$, as would be expected from active T-cell:APC interactions. CD8 + T-cells can have a minor CD4 signal not expected to co-localize with CD3 in CD8 + cells, providing a control. Consistent with these expectations, the correlation of $\sigma_{CD3}$ and $\sigma_{CD4}$ (r=0.17) in CD8 + cells is less than that between $\sigma_{CD3}$ and $\sigma_{CD8}$ (Fig. 1C). Likewise, for CD4 + T-cells, we observed a strong correlation of CD3 and CD4 toward adjacent APCs ($\sigma_{CD3}(\;\text{T-CD4}+,APC)$ and $\sigma_{CD4}\;(\;\text{T-CD4}+,APC)$, r=0.56), consistent with detection of TCR-mediated synapses. As expected, the correlation of $\sigma_{CD3}(\;\text{T-CD4}+,APC)$ and $\sigma_{CD8}(\;\text{T-CD4}+,APC)$ is lower (r=0.26) than the CD3:CD4 correlation.

While our results show that colocalization of TCR proteins toward APCs can be detected by CISA, some protein colocalization might occur regardless of an APC contact. To evaluate the contact-independent effect, we calculated pixel-wise correlations between CD3 and CD4 (or CD8) intensity within the complete T-cell areas in each spot. CISA scores exhibited stronger correlations than the contact-naïve pixel-wise methods (Fig. 1D). This was observed for correlations of CD3 and CD8 in CD8 + T cells (t-test, p=5.8e-9), as well as for correlations of CD3 and CD4 in CD4 + T cells (t-test, p=3.9e-7). These results indicate that CISA captures information on contact-dependent synapse activity.

To verify the robustness of these results, we analyzed a second IMC dataset from Hoch et al.^34^ It consists of 167 tissue microarray (TMA) spot images from melanoma patients. This dataset showed CISA behaviors consistent with the Moldoveanu et al dataset^35^. CD3 and CD8 CISA correlations in CD8 + T cells were strong (r=0.57, Fig. 1E) and higher than CD3-CD4 CISA correlations in the same cells (r=0.09). CD3 and CD4 CISA correlations in CD4 + T cells were also strong (r=0.43, Fig. 1E), and higher than CD3-CD8 CISA correlations in those cells (r=0.14. As in the Moldoveanu et al dataset, CISA correlations were stronger than pixel-wise correlations (Fig. 1F), both for CD3:CD8 in Tc cells (p=1.8e-12) and for CD3:CD4 in Th cells (p=3.8e-12). We also analyzed the correlations of T-cell:APC CISA scores for all pairs of proteins in the IMC data (**Fig. S1**). These showed a rich clustering structure reflecting behaviors such as TCR co-occurrence, T-cell specificity, and APC-specificity.

**Figure 2:**
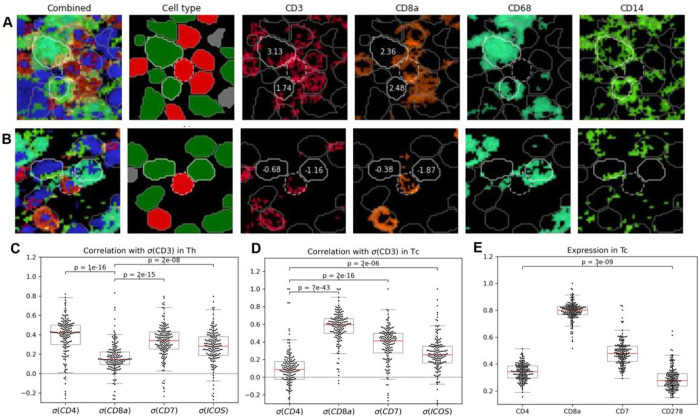
CISA robustly characterizes protein localization at cell-cell interfaces from IMC data. (A, B) Examples of positive (A) and negative (B) localization of CD3 and CD8a around the central T cell (dashed line) toward neighboring macrophages (white line). Numbers indicate synapse strengths. Image width is 50 mm. Cell colors correspond with Fig. 1B. Blue: DNA. Grey outlines: non-contacting or non-APC cells. (C) Correlations of σ(CD4), σ(CD8a) σ(CD7) and σ(ICOS) with σ(CD3) in CD4+ T cells. Each point is one TMA core. (D) Correlations of σ(CD4), σ(CD8a) σ(CD7) and σ(ICOS) with σ(CD3) in CD8+ T cells. (E) Average pixel intensity of markers within CD8+ T cells. ICOS (CD278), is significantly lower than CD4. Each point is the average in a TMA core. (A-B) data are from Moldoveanu et al.35 IMC data. (C-E) data are from Hoch et al.34 IMC data.

To clarify the observed behaviors, example images of T-cell:APC contacts with positive or negative CISA scores are shown in Fig. 2A and B. These images show the variability in signal along membrane boundaries, supporting the importance of statistical approaches that integrate information along over entire cells and tissue images to provide robustness. For example, in addition to CD3, CD4 and CD8a, we were also able to evaluate correlations in CISA scores between CD3 and other T cell surface proteins, including ICOS (CD278) and CD7 (Fig. 2C, D). It is worth noting that ICOS has a significantly (p=3e-9) lower expression level than CD4 in CD8 + T cells (Fig. 2E), but the correlation between $\sigma_{CD3}(\;\text{T-CD8}+,\;APC)$ and $\sigma_{CD278}(\;\text{T-CD8}+,\;APC)$ (Fig. 2D) is stronger than between CD3 and CD4 in those cells. This suggests CISA can detect polarization of cell membrane proteins even at a low expression level. Together, these results support CISA as a robust method to quantify immune synapses between T cells and neighboring APCs.

### Whole-slide images enable regional analysis of tumor microenvironmental interactions

To further verify the applicability of CISA to other types of image data, we generated and analyzed histocytometry ^37^ whole slide images of metastatic melanoma ^38^. These WSIs are much larger than TMA spots and are based on fluorescence, providing substantial differences from IMC. We analyzed a cohort of 21 human metastatic melanoma samples from 20 patients (see Methods). A 6-marker panel was employed to characterize cell phenotypes across whole-slide sections, providing extensive data for identification of TME features and interactions. The images in our cohort averaged 67 mm^2^ of tissue imaged at a resolution of 663 nm per pixel.

**Figure 3:**
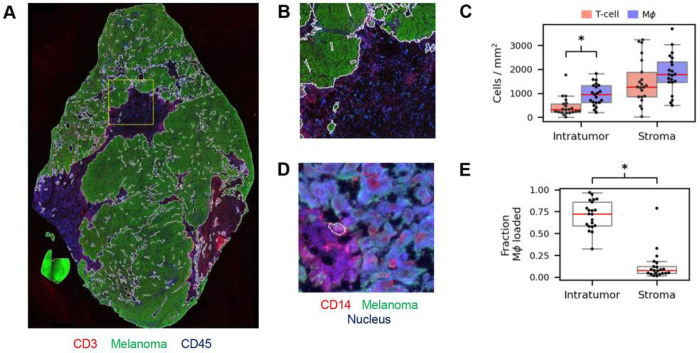
Histocytometryprofiling of immune infiltrate in metastaticmelanoma. (A) An example whole-slide histocytometryimage with select markers shown. White borders denote tumor-stroma interface. Yellow box denotes the zoomed in region for panel B. (B) Zoomed in view of tumor/stroma segmentation. (C) Comparison of immune infiltrate in tumor and stroma. Within the tumor, macrophages are denser than T-cells, but the difference is not statistically significant in the stroma. * denotes p<0.05. (D) An example of a melanoma antigen-loaded macrophage (white bound cell) within the tumor. Note green fluorescence (melanoma antigen stain) within the cytoplasm. (E) Melanoma antigen-loaded macrophages make up the bulk of intratumor macrophage infiltrate (fraction loaded, median = 72.3%), but a significantly smaller fraction in the stroma (median = 7.7%, p=9.5×10-7).MΦ: macrophage. CD3: T-cell. CD14: Macrophages. CD45: Leukocytes.

We first analyzed regional cell type prevalence and spatial co-occurrence in these datasets, as cellular composition ^39^ and APC expression ^38^ could vary between the tumor stroma and tumor nests. The large size of WSI images makes them better than TMA spots for investigating potential region-specific T-cell interactions. After segmenting images into intratumor and stromal regions (see Methods, Fig. 3A, B), we observed that T-cells and macrophages are both abundant in the stroma, but macrophages make up the bulk of the intratumor immune infiltrate. Within the tumor, the cohort median density of T-cells is 86.3 cells per mm^2^, while the density of macrophages is 252.6 cells per mm^2^ (macrophage > T-cell: $p=2.4\times 10^{-4}$, Fig 3C). Macrophages also exhibit differential melanoma antigen loading between the intratumoral and stromal regions, where loading is defined by the presence of melanoma antigen in the cytoplasm (Fig. 3D, see Methods). In the intratumoral region, the majority of macrophages are loaded with unprocessed melanoma antigen (Fig. 3E, cohort median = 72.3%). In the stroma, a significantly lower fraction of macrophages is loaded (cohort median = 7.7%, $p=9.5\times 10^{-7}$).

### T-cells colocalize with macrophages in a region-dependent manner in melanoma

Because of the differential antigen-loading of macrophages between the intratumoral and stromal regions, we hypothesized that cells of the TME interact in a region-specific manner. We investigated this using a radial distribution function (RDF) analysis to identify spatial relationships between cell types in the TME (**Fig. S2A,B**). We applied RDF analysis to quantify the distance-dependent density of TME cells relative to T-cells (see Methods). We additionally devised a metric to quantify T-cell/TME cell colocalization relative to null expectations, ΔCDF, defined from the RDF curve as the excess of colocalization relative to a label-permuting null model – an approach that controls for variations in local cell density (**Fig. S2C**).

**Figure 4:**
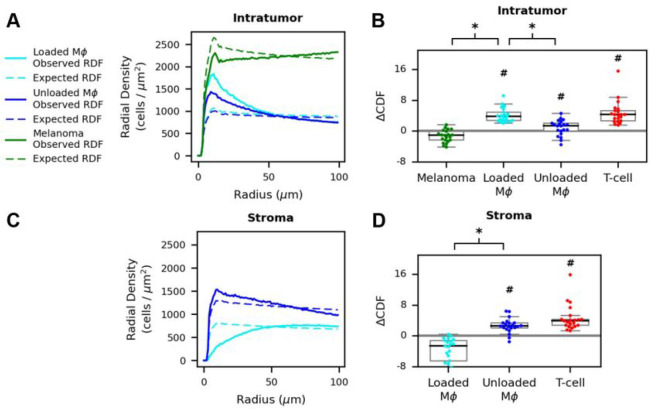
The Radial Distribution Function demonstrates region-specific T-cell interactions with macrophages in whole slide images. (A) RDFs for sample Mel-512 with T-cells as the reference cell demonstrate that intratumor T-cells colocalize with both loaded macrophages (cyan curve, left) and unloaded macrophages (blue) above the expected distribution (dashed lines). Conversely, intratumor T-cells contact melanoma cells below null expectations (green). (B) The cohort distributions of ΔCDF demonstrate significant intratumor T-cell colocalization with loaded macrophages, unloaded macrophages, and other T-cells but not tumor cells. ΔCDF is significantly greater for loaded macrophages compared to both unloaded macrophages and melanoma cells. (C) In the stroma, T-cells colocalize with unloaded macrophages (blue, right) but no colocalization with loaded macrophages. (D) Cohort wide ΔCDF demonstrates significant intratumor T-cell colocalization with unloaded macrophages and other T-cells but not loaded macrophages. ΔCDF for stromal T-cells is significantly greater for unloaded macrophages compared to loaded macrophages. T-cells similarly show strongly positive ΔCDF.MΦ: macrophage. #: 1-sample t-test p<0.05. *. Wilcoxon rank-sum test p<0.05.

The use of RDF analysis to reveal cell-cell spatial associations is illustrated in Fig. 4 for sample Mel-512, with T-cells as the reference cell. In the intratumoral region, a peak in the RDF for loaded macrophages at approximately 12 μm indicates that T-cells and loaded macrophages are often in close proximity (Fig. 4A, solid cyan line). The observed distribution is above the cell-permuted null expectation (Fig. 4A, dashed cyan line) indicating excess colocalization. T-cell colocalization with loaded macrophages is significant when calculated over the full histocytometry cohort ($\Delta \bar{\text{CD}}F=4.184$, 1-sample t-test $p=9.6\times 10^{-10}$, Fig. 4B). Unloaded macrophage colocalization with intratumoral T-cells is also observed (Fig. 4A), though the effect is weaker than for loaded macrophages. Such colocalization is moderately significant across the cohort ($\Delta \bar{\text{CD}}F=0.908$, 1-sample t-test p=0.026, Fig. 4B). Tumor cells have high absolute densities near intratumoral T-cells, but do not significantly colocalize ($\Delta \bar{\text{CD}}F=-1.311$, 1-sample t-test p=1.0, Fig. 4A,B). Intratumor T-cell colocalization with loaded macrophages is significantly greater than with either unloaded macrophages or tumor cells (Fig. 4B, $p=1.2\times 10^{-4}$ and 5.9×10^−5^ respectively).

In the stromal regions, cell co-localization relationships differ from those within the tumor. For example, in the stroma of sample Mel-512, unloaded macrophages have a small RDF peak with respect to T-cells at a distance of approximately 10 μm ((Fig. 4C, blue). This colocalization exceeds the null expectation by a statistically significant but small margin ($\Delta \bar{\text{CD}}F=2.642$, 1-sample t-test $p=1.5\times 10^{-6}$, Fig. 4D). On the other hand, loaded macrophages have lower colocalization with stromal T-cells than expected (Fig. 4C).

Negative colocalization between these cell types is observed across the cohort ($\Delta \bar{\text{CD}}F=-3.567$, 1-sample t-test p=1.0, Fig. 4D). The unloaded macrophage ΔCDF is significantly greater than for loaded macrophages (p=5.9×10-5). Meanwhile, T-cells exhibit strong colocalization to other T-cells both in the stroma and within the tumor ($\Delta \bar{\text{CD}}F=4.616$ and 4.713, 1-sample t-test $p=1.8\times 10^{-6}$ and $4.7\times 10^{-7}$, stroma and intratumor respectively Fig. 4B, D; **S2B**).

### CISA reveals region-specific T-cell/macrophage synapses in whole slide images

**Figure 5:**
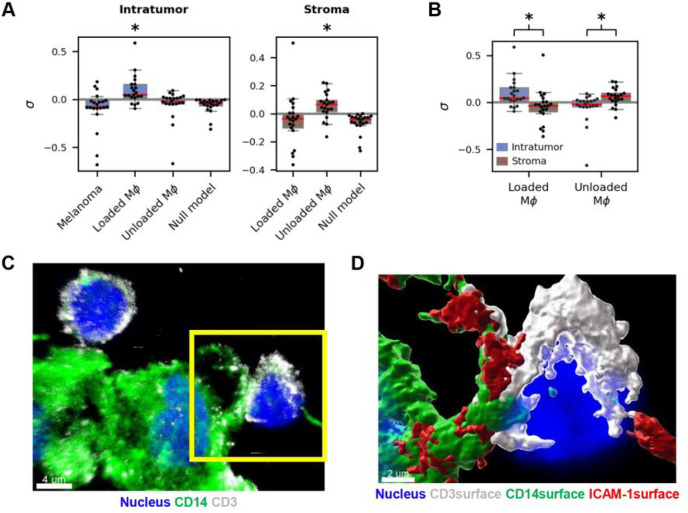
Functional synapse interactions in metastatic melanoma. (A) Intratumor T-cell synapses with loaded macrophages are significantly stronger than those with unloaded macrophages and melanoma (p=3.7×10-4 and p=6.9×10-5 respectively) and are also stronger than expected from the intratumor null synapse model (denoted by *, p=2.4×10-5). Stromal T-cell synapses with unloaded macrophages are significantly stronger than those with loaded macrophages (p=1.3×10-3) and the stromal null model (denoted by *, p=2.9×10-6). (B) Intratumor T-cell synapses with loaded macrophages are stronger than stromal T-cell synapses to loaded macrophages (p=6.9×10-5). In contrast, T-cell synapses with unloaded macrophages are stronger in the stroma than in the tumor (p=1.3×10-3). (C) Super-resolution imaging of two stromal T-cells in contact with CD14+ cells in the TME. The T-cell on the right is in contact with the neighboring CD14+ cell’s dendrite, and CD3 is aggregating in the contact region. Scale bar = 4 μm. (D) Volumetric rendering of the right T-cell in D with additional ICAM-1 rendering. The CD14+ cell’s ICAM-1 can be seen aggregating in the dendrite. Scale bar = 2 μm. APC: antigen-presenting cell. σ: sample-level synapse strength. MΦ: macrophage.

Given the distinct cell co-localization behaviors in the intratumor and stromal regions, we hypothesized that CISA analysis would detect regional differences in immune synapse formation within whole slide histocytometry images. Indeed, CISA analysis of intratumoral regions showed that intratumor T-cells tend to form synapses to loaded macrophages ($\overset{‾}{\sigma }=0.098$, 1-sample t-test $p=3.9\times 10^{-3}$, Fig. 5A), consistent with the IMC results. However, this relationship was not observed in the stroma, and stromal T-cells instead tend to form synapses to unloaded macrophages ($\overset{‾}{\sigma }=0.059$, 1-sample t-test $p=5.0\times 10^{-3}$). Both synapse strengths are significantly greater than the null model ($p=2.4\times 10^{-5}$ and $2.9\times 10^{-6}$ respectively). T-cell synapses to loaded macrophages are significantly stronger within the tumor than in the stroma, while T-cell synapses to unloaded macrophages are significantly stronger in the stroma than in the tumor (Fig. 5B, $p=6.9\times 10^{-5}$ for both respectively). Within the tumor, T-cell synapse strength to loaded macrophages is significantly stronger than to unloaded macrophages (Fig. 5B, $p=3.7\times 10^{-4}$). In the stroma, T-cells have significantly stronger synapses to unloaded macrophages than to loaded macrophages $\left(p=1.3\times 10^{-3}\right)$. In the tumor, T-cells showed no tendency to form synapses to melanoma cells ($\overset{‾}{\sigma }=-0.101$, 1-sample t-test p=0.98), with synapse strength not significantly greater than the null model (p=0.41, Fig. 5A). The greater T-cell synapse strength to loaded macrophages than to melanoma cells is also statistically significant $\left(p=6.9\times 10^{-5}\right)$.

We employed synapse-focused super-resolution imaging to further verify T-cell synapses in the TME. Figure 5C shows two T-cell-macrophage contacts with different CD3 aggregation behavior via stimulated emission depletion microscopy (Fig. 5C). The left T-cell exhibits relatively homogenous CD3 along its membrane without aggregation towards its neighboring macrophage. Volumetric rendering suggests a CD3-containing T-cell protrusion towards the macrophage which may signify early synapse formation or antigen sampling (**Fig. S3A**), though there is not a reciprocal concentration of ICAM-1 in the macrophage that would demonstrate mature synapse formation (**Fig. S3B**). The right T-cell shows CD3 concentration towards the dendrite protrusion on the target macrophage (Fig. 5C, yellow box). Volumetric rendering shows not only contact between the T-cell CD3 and the macrophage dendrite but also reciprocal macrophage ICAM-1 in the contact area, as expected from mature synapse formation and functional interaction (Fig. 5D, **Fig. S3B**).

### T-cell-macrophage interactions are associated with T-cell proliferation in metastatic melanoma

**Figure 6:**
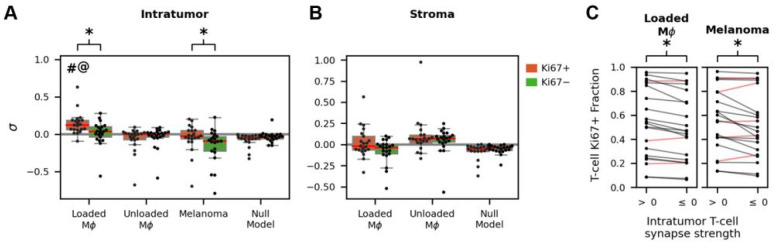
Proliferative consequences of the T-cell-macrophage interaction in metastatic melanoma. (A) Proliferative (Ki67+, orange boxes) T-cell synapses are significantly stronger than non-proliferative (Ki67-, green boxes) T-cell synapses, for both loaded macrophages and melanoma cells (p=4.2×10-4 and p=0.021 respectively). Proliferative T-cell synapses with loaded macrophages are stronger than those with unloaded macrophages (#,p=6.0×10-5) and the null model (@,p=9.5×10-7). (B) In the stroma, there are no significant differences in synapses between proliferative and non-proliferative T-cells with either loaded or unloaded macrophages. (C) Paired difference plot comparing the fraction of intratumor Ki67+ T-cells vs. synapse strength. The fraction of T-cells that are Ki67+ is significantly greater for T-cells with positive synapses compared to non-positive synapses, both for synapses to loaded macrophages (p=2.8×10-4) and to melanoma cells (p=0.014). Black lines indicate a positive difference while red lines indicate a negative difference. σ: sample-level synapse strength. MΦ: macrophage.

T-cells recognizing their cognate antigen in conjunction with pro-inflammatory signals undergo clonal expansion ^40^ typically within the lymph node, but *in vivo* mouse data suggest this may occur in the tumor as well ^15^. We therefore hypothesized that proliferating T-cells in the TME might have stronger synapse strengths than other T-cells. We extended CISA to address this by classifying T-cells as proliferating (Ki-67+) or non-proliferating (Ki-67−) from KI-67 imaging data. We found that intratumor Ki-67 + T-cells have significantly stronger synapses to loaded macrophages than Ki-67 − T-cells (Fig. 6A, $p=4.2\times 10^{-4}$) and significantly greater than the Ki-67 + T-cell null model $\left(p=9.5\times 10^{-7}\right)$. There was no significant difference in synapse strength between Ki-67 + and Ki-67 − stromal T-cells, unlike in the intratumor region (Fig. 6B). Ki-67 + T-cell synapse strength to melanoma cells is also stronger than for Ki-67 − T-cells (p=0.021), although not significantly greater than the null model. To further address the synapse-proliferation association, we compared the fraction of T-cells that are Ki-67 + in the synapse positive and non-positive groups. For intratumoral T-cells in contact with loaded macrophages, we observed a significantly greater fraction of Ki-67 positivity in synapse-positive T-cells compared to non-positive synapse T-cells ($p=2.8\times 10^{-4}$, Fig. 6C). We observed a similar association between KI-67 and T-cell/melanoma synapses, albeit with lower statistical significance (p=0.014).

On the other hand, T-cell synapse formation with unloaded macrophages was not associated with a proliferative response. We observed no significant difference in average synapse strength between Ki-67 + and Ki-67 − T-cells in contact with unloaded macrophages, either within the tumor or in the stroma (Fig. 6A, B). Additionally, intratumoral Ki-67 + T-cells have significantly higher average synapse strengths with loaded macrophages than with unloaded macrophages (Fig. 6A, $p=6.0\times 10^{-5}$). In the stroma, T-cell Ki-67 status is not associated with synapse strength to an adjacent macrophage, regardless of whether the macrophage has an antigen load.

### Synapse analysis of breast cancer imaging mass cytometry data reveal T-cell/B-cell interactions

To test the applicability of the RDF and CISA approaches to other types of tumors, we applied these methods to a cohort of breast cancer IMC images of primary tumors from 281 patients ^25^, of which 275 had associated survival data and clinical subtyping. Because these IMC images were derived from tissue microarrays, individual IMC spot images had far fewer cells than histocytometry images, resulting in noisy and uninformative sample-level T-cell RDF curves. Consequently, we aggregated all breast cancer IMC images to generate a single cohort RDF for each class of cell-cell co-localizations. We did not distinguish antigen-loaded from -unloaded macrophages in this analysis because loaded macrophages make up only 0.3% of macrophages in the stroma and only 11% of the total macrophage content.

RDF analysis showed no distinguishable colocalization of T-cells with tumor cells within breast tumors (ΔCDF=-0.465, **Fig. S4**), similar to T-cells within melanomas. This behavior was consistent across clinical subtypes, with HER2 + tumors showing the largest (negative) deviation from the null (ΔCDF=-2.260, **Fig. S5A-C**). Intratumor T-cell/macrophage associations were close to null expectations (ΔCDF=0.509). T-cell/B-cell interactions were below null expectations, though noisy. This is related to the fact that only 2.6% of B-cells reside within tumor nests, which hinders statistical assessment of colocalization. In the stromal region, T-cells show a co-localization with both macrophages and B-cells, and these effects are of comparable magnitude (ΔCDF=1.261 and 1.567 respectively). The T-cell co-localization with B-cells is apparent despite the presence of B-cells in only 130 of 275 images, compared to 272 patient images containing macrophages. Stromal T-cell colocalization with B-cells and macrophages is consistent across clinical subtypes with some variation in effect size (**Fig. S5A-C**).

**Figure 7:**
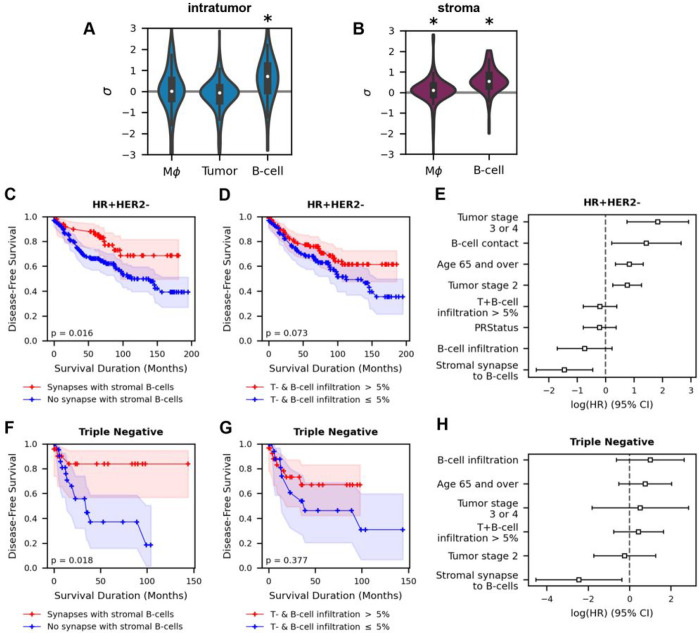
T-cell synapses to B-cells are associated with breast cancer disease-free survival. (A) Immune synapses of T-cells to B-cells are significantly >0 in tumor nests (denoted by $\overset{‾}{\sigma }=0.839$, 1-sample t-test p=5.5×10-5), (B) and in the stroma ($\overset{‾}{\sigma }=0.601$, 1-sample t-test p=6.8×10-16). There is a significant but low effect size sfor T-cells to macrophages in the stroma. (C) HR+HER2− patients with stromal T-cell synapses to B-cells have significantly improved disease-free survival (DFS) in Kaplan-Meier (KM) survival analysis (p=0.016). (D) Patients with increased immune infiltration do not have significantly improved DFS (p=0.073). (E) Cox proportional hazards (PH) shows that stromal synapses to B-cells have a significantly negative log hazard ratio (HR) in HR+HER2− patients when compared to other infiltrative and clinical factors (logHR=-1.45,p<0.005) in determining DFS. (F) Triple-negative breast cancer patients with stromal T-cell synapses to B-cells have significantly improved DFS in Kaplan-Meier (KM) survival (p=0.018). (G) Patients with increased immune infiltration do not have significantly improved DFS (p=0.377). (H) Cox proportional hazards (PH) shows the stromal synapses to B-cells have a significantly negative log HR in triple-negative patients when compared to other infiltrative and clinical factors (logHR=-2.45,p=0.02). σ: sample-level synapse strength. $\overset{‾}{\sigma }$: cohort-level mean σ.

We then applied CISA to the breast cancer IMC images to investigate T-cell synapses with macrophages and B-cells. In the intratumoral region (Fig. 7A), T-cells do not have significant synapse formation to macrophages ($\overset{‾}{\sigma }=0.078$ 1-sample t-test p=0.28) or tumor cells ($\overset{‾}{\sigma }=-0.235$, 1-sample t-test p=1.0), consistent with the lack of T-cell/cancer synapses in melanoma. A noteworthy effect in the breast IMC images is that intratumor T-cells form strong synapses with B-cells ($\overset{‾}{\sigma }=0.839$, 1-sample t-test $p=5.5\times 10^{-5}$). These effect sizes are larger than those for the T-cell/loaded macrophage interactions in melanoma (Fig. 5A, 5B, 6A, 6B). Remarkably, this synapse effect is highly significant despite the lack of frequent colocalization between B-cells and T-cells – intratumor T/B-cell contacts occur in only 46 of 275 (17%) images. In the stroma, T-cell synapse behaviors are consistent with the tendency of both macrophages and B-cells to co-localize with T-cells. Stromal T-cells have significant synapse formation with macrophages ($\overset{‾}{\sigma }=0.088$, 1-sample t-test p=0.035, Fig. 7B), on par with the effect size seen in melanoma (Fig. 5A). Moreover, stromal T-cells form even stronger immune synapses with B-cells ($\overset{‾}{\sigma }=0.601$, 1-sample t-test $p=6.8\times 10^{-16}$). Curiously, due to the paucity of B-cells, stromal T-/B-cell contacts are present in only 104 of 275 (38%) patients, compared to macrophage contacts being present in 235 patients. Synapse behaviors do not significantly differ across breast cancer subtypes (1-way ANOVA p>0.05 for each target cell, **Fig. S5D-F**).

### Stromal T-cell synapses with B-cells are associated with improved breast cancer survival

B-cells can hinder or help tumor growth depending on the context, and prior studies have suggested T-cells may be necessary for the beneficial B-cell effects ^41^. We therefore tested whether the T-cell synapses to B-cells were associated with clinical outcomes in breast cancer. We first analyzed the 173 hormone-receptor positive HER2 negative (HR + HER2−) patients. We divided these patients into two groups based on synapse formation to B-cells: 1) those with a stromal T-cell:B-cell σ>0 (representing contact and synapse formation), vs. 2) those with either σ<=0 (representing contact with no synapse formation) or no contact (and therefore no synapse formation) between T- and B-cells. We observed a significant difference in disease-free survival (DFS) between these groups by Kaplan-Meier (KM) estimation, with improved survival for group 1 (p=0.016, Fig. 7C). This DFS benefit is specific to the stroma, as splitting by the intratumor σ does not result in a significant survival difference (p=0.88, **Fig. S6A**), nor does splitting by the combined intratumor and stromal T-cell σ(p=0.106, **Fig. S6B**). DFS differences cannot be explained simply by contact between stromal T- and B-cells: grouping patients by the presence or absence of T-cells in contact with B-cells regardless of synapse strength resulted in a non-significant DFS difference (p=0.131, **Fig. S6C**). Additionally, grouping patients by the proportion of T- and B-cell infiltration was not sufficient for a significant difference in DFS (p=0.073, Fig. 7D). We next integrated these factors into a multivariable survival regression model using Cox Proportional Hazards, regressing the aforementioned factors and patient metadata against DFS. Stromal T-cell synapse formation with B- cells exhibited a negative and significant hazard ratio (logHR=-1.45,CI[-2.45,-0.45], p<0.005, Fig. 7E) in distinction from other factors, including increased immune cell infiltration.

We next investigated the association of DFS with T-cell/B-cell interactions in other breast cancer molecular subtypes. The Jackson et al. cohort^25^ has 48 TNBC (HR - HER2−) and 52 HER2+ (including 29 HR + and 23 HR−) patients. Splitting patients again by stromal T-cell σ to B-cells, the survival benefit in TNBC is significant by KM survival estimation (p=0.018, Fig. 7F). No samples had a non-positive sample-average synapse strength in the TNBC cohort, either when considering only stromal or both stromal and intratumor synapses. As such, we could not test for additional survival benefits of T-cell-B-cell contact. Survival was not significantly different when splitting patients by quantity of T- and B-cell infiltration (p=0.377, Fig. 7G). Multivariable survival regression demonstrated T-cell synapse formation to B-cells has a strong effect on DFS after controlling for infiltration factors (logHR=-2.45,CI[-4.52,-0.38], p=0.02, Fig. 7H). In the HER2 + cohort, KM survival estimation shows no DFS benefit for stromal T-cell synapse formation to B-cells (p=0.727, **Fig. S6D**) or increased T- and B-cell infiltration (p=0.515, **Fig. S6E**). Rather, the presence of hormone receptors estrogen receptor (ER) and progesterone receptor (PR) are the factors influencing survival in the Cox Proportional Hazards model with opposite effects (ER: logHR=1.56,CI[0.59,2.72], p=0.01, PR: logHR=-1.72,CI[-3.02,-0.41], p=0.01, **Fig. S6F**). Thus, T-cell synapses to B-cells have strong survival benefits in TNBC but not HER2 + patients in this cohort.

## Discussion

Image analysis of *in situ* human tumor samples can deepen our understanding of the functional and compositional heterogeneity within the TME. Here we have presented CISA, a novel algorithm to quantify T-cell immune synapses by analyzing the distribution of proteins at cell-cell interfaces in multiplexed images of tumor tissues. We have verified the effectiveness of CISA across multiple imaging mass cytometry datasets in melanoma and breast cancer and further validated it with whole slide histocytometry images and super resolution emission depletion microscopy images generated by our team. Using CISA, we were able to elucidate many aspects of T-cell synaptic interactions, including the quantification of T-cell:macrophage interactions in melanomas, the increased proliferation of T-cells forming synapses with macrophages, and the differential behavior of T-cell:macrophage interactions between tumor and stromal regions. To our knowledge, CISA is the first computational method able to mine high resolution imaging data to quantify immune synapses. While we have focused on TCR proteins, CISA can handle any synapse-associated molecules. For example, CISA could be used to study other clinically important synapse-mediated mechanisms such as reciprocal PD-1/PD-L1 engagement at immune synapses ^42,43^ and their downstream effects on TCR engagement strength ^44^.

Our analysis of a large breast cancer cohort highlights the flexibility and potential clinical utility of CISA. We discovered that CISA quantification of T-cell: B-cell synapses is predictive of improved survival. Remarkably, this predicted benefit is beyond what would be expected simply from lymphocyte infiltration 3, particularly in HR + HER2 − and triple-negative breast cancer tumors. These findings are notable given the small sizes of TMA spots, the rarity of B-cells, and the small number of immune markers in the IMC data, suggesting that the scale of data considered here will be sufficient for further synapse biology discoveries.

The T-cell synaptic associations we have observed are relevant to a number of prior studies. For example, enhancement of macrophage tumor cell phagocytosis with anti-CD47 antibodies has been shown to yield tumor regression ^45–48^ in a T-cell-dependent manner ^49^. Our observation that T-cells form synapses with melanoma-loaded macrophages suggests that macrophage phagocytosis of tumor cells impacts priming and proliferation of T-cells to maintain anti-tumor response. B-cells are known to exert and promote pro-or anti-tumor immunity in a context-dependent fashion ^41,50^. T-cells may be necessary for the beneficial B-cell effects ^41^ and vice versa ^51^, including within the context of immunotherapy ^52–54^. Our results support that these behaviors stem from the enhanced activation of anti-tumor T cell responses via synapses with antigen-presenting B cells ^55^. Differences in B-cell infiltration may explain the lack of observed effects in melanoma compared to primary breast tumors^56,57^

We expect that CISA analyses will improve as multiplexed tumor imaging with broader marker panels increase, as these will enable finer interrogation of synaptic protein co-localization patterns. Multiplexed imaging approaches are limited by the number of concurrent assayable markers ^37^ or capture duration ^58^, and current panel sizes necessitate tradeoffs among tumor and immune proteins. This make it difficult to resolve drivers of spatial effects such as the proliferation observed in the melanoma histocytometry data. Larger marker panels would also refine understanding of how specific T-cell populations such as Tregs ^59^ contribute to prognosis. 3-dimensional imaging ^60^ is also likely to improve the quantification of immune synapses and their impact on tumor cell killing in time and space ^61^.

A caveat of CISA is that it depends on the quality of cell segmentation to accurately identify membrane pixels. We showed that melanoma datasets imaged and segmented by distinct approaches yielded similar synaptic behaviors when analyzed with CISA, but there remain uncertainties due to variations in data characteristics and associated segmentation approaches. For example, the cell segmentations from our histocytometry cohort were based on Imaris’ proprietary algorithms. In the breast cancer cohort, segmentations were generated by the combination of Ilastik ^62^ and CellProfiler ^63^, a common protocol but potentially dependent on manual tuning. Machine-learning segmentation techniques such as StarDist ^64^, Cellpose ^65^, and MESMER ^66^ offer high-throughput options that should improve as more publicly-available datasets emerge. A related consideration is that cell classification methods also vary, e.g. cell classification schemes designed specifically for single-cell mass cytometry applications such as X-shift ^67^ may be important to optimal analysis of IMC data. Nevertheless, the reason that CISA is able to yield robust results in spite of such uncertainties is likely because it integrates over the many cell-cell interactions within a TMA spot or whole slide image, clarifying the signal.

In conclusion, CISA is a powerful approach capable of capturing region-specific T-cell synaptic interactions in the TME associated with biological and clinical outcomes. Moreover, it can in principle be applied to any highly-multiplexed epitope imaging modalities. We expect that CISA analysis will be valuable for understanding the functional and clinical correlates of specific T-cell interactions to guide future therapies.

## Methods

### Metastatic melanoma samples for histocytometry

De-identified metastatic melanoma surgical samples were obtained from the Cooperative human tissue network (CHTN) or from the Baylor Research Institute Tissue Bank. The use of these samples was determined to be non-human subjects research by the Jackson Laboratory Institutional Review Board. Resection location was provided but other clinical data such as past treatment was incomplete. No patient outcomes were available as no follow-up was allowed per the agreement with CHTN. Additional information provided in Martinek et al., 2022^38^.

### Tissue Immunofluorescence Staining

Optimal cutting temperature compound (OCT) embedded samples were cryo-sectioned (8um) and consecutively, fixed with acetone, washed with 1x PBS, treated with Fc Receptor Block (Innovex bioscience) for 40 min + Background Buster (Innovex bioscience) for an additional 30 min. The sections were then stained with primary antibodies, diluted in PBS + 5% BSA 0.1% Saponin for 1 hour at room temperature, washed and stained with the secondary antibodies at room temperature for 30 minutes, secondary antibodies were saturated using mouse normal serum (Jackson ImmunoResearch) for 15 minutes at room temperature. Tissues were stained with directly conjugated antibody mix for 1 hour at room temperature and washed. Nuclei were counterstained with 4’,6-diamidino-2-phenylindole (1ug/mL) or SytoxBlue 1/1000 for 2 minutes. Tissues were mounted in Fluoromount-G mounting media.

Primary anti human antibodies: CD3 (HIT3a, Biolegend); CD14 (UCHM1, Genetex); MART-1 (M2-7C10 + M2-9E3 Novus Biologicals); PMEL (NIKI/beteb LSBio).

Conjugated anti human antibodies: CD3-BV421 (UCHT1, Biolegend); CD45-AF488 (HI30, Biolegend) CD19-AF700 (HIB19, Biolegend); CD138-AF700 (MI15, Biolegend); Ki67-AF555 (MKI67, BD Pharmingen); ICAM-1-APC (HA58, Biolegend); Melanoma-JF549 (M2-7C10 + M2-9E3 + T311 + HMB45, Novus Biologicals).

Secondary antibodies: depending on primary antibody combinations, species and isotype specific secondary antibody conjugated with either Alexa Fluor 488, Alexa Fluor 568 and Alexa Fluor 647 (Molecular Probes) were used in appropriated mixtures.

### Whole section scan

Whole tissue scans were acquired on the Leica SP8 confocal microscope (Leica Microsystems) equipped with an automated motorized stage. Sequential acquisition was performed with a 20X objective. For each tile, focal plane was defined by autofocus function based on nuclear staining. Tiles were max projected and stitched using Leica LAS X software.

### Super resolution microscopy (3D STED)

Tissue sections were stained following a modified immunofluorescence staining protocol with 2-fold increase in antibody concentration and one additional wash at each washing step. Vector Shield mounting media was left to cure for 72 hours. Super resolution acquisition was performed on an inverted Leica SP8 confocal microscope equipped with STED modules, with 3 depletions lasers and an HC PL APO 100x GLYC objective (Leica Microsystems). Z-stacks were acquired with the 3D STED function using the 775nm depletion laser. Images were analyzed and surface rendering was performed using the Imaris software (Bitplane).

### Histocytometry

*In situ* quantitative analysis of melanoma tissue was based on published methodology ^37,68,69^. Briefly, whole tissue scans were acquired using a Leica SP8 confocal microscope (Leica Microsystems, Germany). Each scan was then analyzed using image analysis software Imaris 8.4 (Bitplane). Using the “spot” function in Imaris, the images were segmented into individual cells, defined based on having a nucleus diameter equal or larger than 5um, used as a seeding point for each cell’s spot. The accuracy of the segmentation was visually assessed and adjusted if needed for each sample. Finally, for each generated spot, x;y coordinates and the mean intensity values for all channels were exported into an fcs file to be visualized and quantified using Flowjo software (version 10, Flowjo LLC).

### Histocytometry image processing and analysis strategy

Single-cell masks from cell segmentation were exported from Imaris, and processed using the Python package scikit-image ^70^. Small holes in the masks were filled and per marker single-cell average fluorescence intensities recalculated from the modified masks. The one pixel-wide internal mask boundary was considered as the membrane and the remaining pixels as the cytoplasm for additional signal quantification.

We used a K-means-based adaptive thresholding scheme for both tumor/stroma segmentation and cell label assignment in the histocytometry cohort to accommodate for inter-sample signal variation. Tumor/stroma segmentation for each image was conditioned on the signal intensity of the melanoma and CD45 channels respectively. The two image channels were passed through a gaussian filter (σ=1) and ${log}_{2}(x+1)$ transformed, where x represents the pixel value, to prevent negative values. We used Multi-Otsu thresholding, which is equivalent to the K-means clustering of the intensity histograms ^71^, to determine thresholding levels. We considered four clusters which approximate to non-tissue background, tissue background, low intensity foreground, and high intensity foreground. The final signal threshold value was chosen as the mean signal in the low intensity foreground cluster to help limit undersegmentation of the tumor. Intensities greater than the threshold were considered part of the individual mask. Individual masks were furthered processed with a morphological closing (10-pixel square) followed by removing small holes (fewer than 2500 pixels) to eliminate small gaps. Small objects (fewer than 2500 pixels) were also removed to ignore isolated cell debris. We considered the tumor segmentation as the processed melanoma channel mask; the stroma segmentation was the processed CD45 channel mask excluding intersection with the melanoma channel mask. This allows for immune cells which reside in tumor nests to be differentiated from those in the stromal regions of the tumor.

Our K-means clustering approach for cell label assignment considered two clusters, approximating non-membership and membership. We clustered the ${\text{log}}_{2}$-transformed single cell average fluorescence intensities (again biased by + 1) for each channel within a sample separately. To reduce the potential for false positives, we chose the mean of the membership cluster as our threshold to label cells. We modified the quantification of single cell signal intensities to call antigen-loaded macrophages in our histocytometry cohort. Instead of using the entire macrophage area to determine melanoma signal to call antigen-loaded or unloaded, we took the upper-quartile average of the melanoma signal in the cytoplasm mask and determined loaded macrophage membership using the melanoma channel threshold. Our gating strategy allows for membership in multiple classes, such as cells labeled as both CD3+ (T-cell) and CD14+ (macrophage) which should not be expressed in a single immune cell together. However, we observed that these cases were due to undersegmentation of cells such that T-cells in contact with macrophages were pressed up closely enough such that nuclei were indistinguishable for segmentation. This did not affect our spatial analyses significantly but is an artifact to consider that may cause our immune synapse analysis to underestimate the strength of T-cell synapses with macrophages.

### External Imaging Mass Cytometry data analysis

For the IMC data of (Moldoveanu et al, 2022)^35^, the published data include cell center coordinates and cell classification, but no cell segmentation. We performed cell segmentation with Mesmer^66^, then mapped the cell centers to the cell classification of the nearest cells, within a maximum center-to-center distance of 15 pixels. For the IMC data of (Hoch et al, 2022)^34^, cell segmentations were available, but cell classifications were not provided. We calculated the average intensity of markers in each cell, and rescaled the mean intensity to a [0,1] range where values equal or greater to the 99th percentile of all cells were mapped to 1. Then we applied hierarchical clustering with Ward’s method and annotated the top 20 clusters based on known cell type specific marker patterns in the cluster mean intensity. This process yielded cell classifications for major immune and tumor cell types. In Fig. 2G, expression was calculated by averaging pixel intensity within each cell, then averaging the behavior of cells within each TMA.

Data from the breast cancer IMC cohort ^25^ were obtained from https://doi.org/10.5281/zenodo.3518284. Data were minimally processed, as the necessary elements for our RDF and synapse analysis were provided from the publication. This includes spillover-corrected images, tumor/stroma masks, single-cell masks, and cell labels. We used the provided cell labels in our analysis, combining the metaclusters considered epithelial cells into a single tumor cell label for our general analysis. In addition, we combined the two T-cell only metaclusters into a single cluster and the two macrophage metaclusters into a single cluster. We excluded metacluster 2 for B- and T-cell labeling due to the ambiguity. Reclustering only immune cells did not produce meaningful separation in the metacluster to discern T- and B-cells (data not shown).

To mitigate the bias of random single strong pixels in IMC data, all raw images were log-transformed before analyzed by CISA. To avoid inducing additional bias, low intensity pixels were not removed as noise. Such noise is addressed implicitly as CISA compares the contacting region with the non-contacting region.

### CISA correlation analyses

For the correlations in CISA scores (Fig. 1B and Fig. 1D), σ values were computed for all contacts between cells of the two specified types. The figures provide density plots of the σ values. The overall correlation r was evaluated across these σ values. For the pixel-wise correlation analyses (left panels of Figs. 1C and 1E), for each sample all pixels within the area of any segmented T-cells were identified. We then computed the correlation of the intensities for the specified proteins (CD3 vs. CD8; or CD3 vs CD4) over all pixels within the specified T-cell type (Tc or Th). Thus each dot in Figs. 1C and 1E corresponds to a sample. The CISA correlation values in Figs. 1C and 1E were computed analogously to the correlations in Figs. 1B and 1D, but on a sample-specific basis.

### Cell densities and the radial distribution function

Immune cell densities in histocytometry were calculated using the tumor/stroma masks and cell labels. For sample-level densities (Fig. 3C), we took the label count divided by the area of the respective tumor and stroma mask. B-cells were excluded from analysis due to antibody cross-reactivity with tumors cells, limiting our ability to reliably detect B-cells. The radial distribution function (Fig. 4) was calculated using the nearest neighbors algorithm from the Python package scikit-learn ^72^. RDFs describe the density of target particles for a given distance from a reference particle ^73^ (Fig. S2A). RDFs have been used previously to calculate cell-cell separation distances in cell cultures ^74^, but have not been applied to quantify whole-slide TME spatial relationships to our knowledge. We adapted this concept using T-cells as the reference particle, with the other TME cell types assayed as target particles. RDFs quantify the average relationships between cell types, integrating the full information from selected regions. An advantage of RDFs over nearest neighbor statistics is that the RDF can be interpreted as a function of cell-cell distance.

After calculating all neighbors within 100 μm for each T-cell, we collected the frequency of the cell types of interest at distances binned into 1 μm increments. 100 μm is the range at which most RDF curves reached an asymptotic density; further distances did not yield additional information. We calculated histograms for both stromal and intratumor T-cells, then normalized to the number of T-cells in each region. Each bin of the histograms was further normalized by the area of the annulus representing each binned distance (Fig. S2A, yellow). This formulation of the RDF differs from the textbook definition in the final normalization step, where a traditional RDF definition further normalizes to the overall particle density ^73^. We chose to omit this step to reflect the true density near T-cells as an additional value to compare spatial behaviors. We modified the final normalization step for the IMC analysis, as the smaller imaging area led to edge effects. For each T-cell, we adjusted the counts in each distance bin by the area of the annulus residing in the image divided by the original area of the annulus. Expected RDF curves were generated using label permutation. As we iterated through each T-cell to aggregate distance histograms for the observed RDF curve, we randomly permuted cell labels within 100 μm 100 times while keeping distances constant. We repeated the original RDF procedure for each permutation and averaged all the permutations together to generate the expected RDF curves for each cell type of interest.

The T-cell RDF can be thought of as a probability distribution function (PDF) of finding a target cell a given distance away from the reference cell. We used this concept in our ΔCDF calculations to determine T-cell spatial associations. RDFs were divided by the sum of RDF distance bins out to 100 μm to generate a pseudo-PDF which were then converted to discrete cumulative distribution functions (CDF).ΔCDF was calculated by summing the differences between the observed and expected CDFs at each of the 100 1-μm bins.

### Immune synapse model

To study immune synapse formation in imaging, we formulated the T-cell synapse strength as a means to quantify TCR localization. A T-cell’s synapse strength with an APC is calculated as the average signal intensity of CD3 at the membrane in contact with an APC divided by the average signal in the noncontact area followed by a ${\text{log}}_{2}$ transformation (Fig. 1A). For a given T-cell, we identify the two pixel-wide internal boundary of the cell mask as the cell membrane. A one pixel-wide internal boundary was used for IMC synapse analysis due to the lower resolution. We use a 2-pixel diamond element for morphological dilation on each single cell mask of cells near T-cells to determine which are in contact. The dilated pixels of the neighboring cell intersecting with the cell of interest’s membrane pixels are considered the contact interface (Fig. 1A). The remaining membrane pixels are part of the non-contact interface. In cases where a T-cell is in contact with multiple cells of the same cell type, the non-contact interface is modified to contain only pixels not considered in contact with that cell type. This model is a simple quantitative improvement to a previous *in situ* approach which requires multiple molecule colocalization to identify qualitative synapses ^75^.

We made adjustments to the synapse strength calculation for IMC images due to high signal-to-noise ratio compared to histocytometry for which our synapse analysis was originally designed. The mean CD3 signal in either the contact and/or non-contact interface was 0 for a non-trivial number of T-cells. Rather than discarding these T-cells from the analysis, we added a noise term to provide a fill-in value in these situations. We estimated this noise term for each sample as the background cell CD3 level, calculated as the average of the CD3 signal intensity in cells not labeled as a T-cell. In cases where the mean signal at the contact interface was 0 but the mean signal in the non-contact interface was less than the estimated noise term, we set the synapse strength to 0.

We devised a null synapse model for our histocytometry cohort to account for potential cell segmentation biases and serve as a control comparison in our synapse analysis. The null synapse model was derived by generating randomly-selected contiguous pixel regions on T-cell membranes. We used a seed-and-grow approach to create contiguous regions from membrane pixels to act as the null contact pixels in our synapse model, with the remaining membrane pixels acting as the non-contact pixels for synapse strength calculations. In the seed-and-grow approach, a pixel was randomly chosen from the membrane as a seed. Neighboring pixels were iteratively added until the region consisted of at least 15 pixels. A cell’s null synapse value was calculated from the average of five randomly seeded null synapses. This process was repeated for each T-cell in contact with another cell, with the null model synapse value for an iteration calculated from the sample average of these cell’s null synapse values. The mean of 100 iterations of sample averages were calculated to generate the final null model synapse value. In our initial histocytometry synapse analysis (Fig. 5), T-cells were split into intratumor and stroma to generate separate null models for each region. We further split these populations by Ki67 positivity for the proliferation analysis (Fig. 6).

### Breast cancer IMC survival analysis

Survival analysis was performed on 275 patients with disease-free survival values and clinical subtyping information in the breast cancer IMC cohort. Two patients with available clinical data were excluded due to their lone B-cell residing on the edge of the image, raising the possibility of misclassification and ambiguity of infiltrate. In the case of patients with multiple images, the first was selected if no T-cell-B-cell contact occurred. If T-cell-B-cell contact occurred in one of the images, that image was chosen. There were no instances where one image had a negative synapse and the other image had a positive synapse. The remaining 273 patients were split into three clinical subtypes based on the clinical metadata provided by the authors: HR + HER2−, HER2+ (both HR + and HR−), and Triple Negative (HR − HER2−).

Patients were further subdivided based on parameters of interest. T-cell-B-cell contact was determined based on our synapse analysis. A σ greater than 0 implies contact between T-cells and B-cells and a tendency to form synapses, whereas a σ less than or equal to 0 implies contact but no tendency to form synapses. Samples with no σ do not have contact between T- and B-cells; contact is thus determined as any value of σ. For Figs. 7E and H, patients were split by the percentage of T- and B-cells compared to total cellular content. We used the sum of cells labeled as T- or B-cells, including the ambiguous label previously excluded from synapse analysis. A 5% threshold was set to differentiate tumors with increased lymphocyte infiltrate. This was chosen as the frequency of samples as the level of infiltrate dropped substantially below this cutoff point and is approximately the median lymphocyte infiltration. Survival analysis was performed using the Python package lifelines (version 0.23.0) implementation of Kaplan-Meier survival estimation and the Cox Proportional Hazards model using default parameters. Tumor stage in the Cox Proportional Hazards analysis is based on the column “PTNM_T” using only the numeric stage.

### Statistics

Wilcoxon signed-rank test p-values are presented for comparisons between cell types and regions unless otherwise specified. When 1-sample t-tests are noted, the one-sided greater-than alternative hypothesis was used with the population mean considered to be 0. Presented p-values are unadjusted. Log-rank test was used to determine Kaplan-Meier survival estimation p-values.

## Data Availability

Data and Code availability Software for CISA analysis has been made available through Github at https://github.com/SamLiu1218/CISA. Histocytometry images will be made available upon publication (likely through Zenodo).
